# 活性导向的化学探针在氨基酸反应活性表征中的应用进展

**DOI:** 10.3724/SP.J.1123.2022.05013

**Published:** 2023-01-08

**Authors:** Jiaying LI, Guosheng WANG, Mingliang YE, Hongqiang QIN

**Affiliations:** 1.沈阳化工大学化学工程学院, 辽宁 沈阳 110142; 1. College of Chemical Engineering, Shenyang University of Chemical Technology, Shenyang 110142, China; 2.中国科学院大连化学物理研究所, 中国科学院分离分析重点实验室, 辽宁 大连 116023; 2. CAS Key Laboratory of Separation Science for Analytical Chemistry, Dalian Institute of Chemical Physics, Chinese Academy of Sciences, Dalian 116023, China

**Keywords:** 基于活性的蛋白质组分析, 化学探针, 药物靶点, 氨基酸反应活性, activity-based protein profiling, chemical probe, drug target, amino acid reactivity

## Abstract

原创药物的研制得益于蛋白质新靶标的发现,而新靶标的发现依赖于高可信度、高通量的药物-蛋白质相互作用分析方法。蛋白质作为生命功能的执行者,其表达量、空间定位与结构差异直接影响药效的发挥。目前,超过85%的蛋白质尚被认为是无法成药的,主要原因是缺少药物分子靶向的空腔以及相应的反应活性位点。因此,基于蛋白质组学层次实现对氨基酸反应活性位点的表征成为原创共价靶向药物设计的关键,也是克服难以成药靶标蛋白问题的关键。近年来,质谱技术的飞速发展极大地推动了基于蛋白质组学技术的药物-靶蛋白相互作用研究。其中基于活性的蛋白质组分析(ABPP)策略是利用活性位点导向的化学探针分子在复杂样品中实现功能状态酶和药物靶标等蛋白质的检测。基于化学探针的开发和质谱定量技术的发展,ABPP技术在氨基酸反应活性表征研究中展现出重要的应用潜力,将助力于药物新靶标的发现和药物先导化合物的开发。ABPP策略主要基于蛋白质的活性特征进行富集,活性探针作为ABPP策略的核心,近年来取得了飞速进展。该文回顾了ABPP策略的发展历程,重点介绍基于广谱活性探针的ABPP技术在多种氨基酸反应活性筛选领域的研究进展,并对其在药物靶点发现中的应用前景进行展望。

## Abstract (en)

The discovery of novel drug targets enhances the development of novel drugs, and the discovery of novel target proteins depends on highly accurate high-throughput methods of analyzing drug-protein interactions. Protein expression levels, spatial localization, and structural differences directly affect pharmacodynamics. To date, >20000 proteins have been discovered in the human proteome by the genome and proteome projects via gene and protein sequencing. Understanding the biological functions of proteins is critical in identifying and regulating biological processes, with most remaining unidentified. Until recently, >85% of proteins were considered undruggable, mainly because of the lack of binding pockets and active sites targeted by small molecules. Therefore, characterization of the reactive sites of amino acids based on proteomic hierarchy is the key to novel drug design. Recently, with the rapid development of mass spectrometry (MS), the study of drug-target protein interactions based on proteomics technology has been considerably promoted. Activity-based protein profiling (ABPP) is an active chemical probe-based method of detecting functional enzymes and drug targets in complex samples. Compared with classical proteomics strategies, ABPP is based mainly on protein activity. It has been successfully utilized to characterize the activities of numerous protease families with crucial biological functions, such as serine hydrolases, protein kinases, glycosidases, and metalloenzymes. It has also been used to identify key enzymes that are closely related to diseases and develop covalent inhibitors for use in disease treatment. The technology used in proteome analysis ranges from gel electrophoresis to high-throughput MS due to the progress of MS technology. ABPP strategies combined with chemical probe labeling and quantitative MS enable the characterization of amino acid activity, which may enhance the discovery of novel drug targets and the development of lead compounds. Amino acid residues play critical roles in protein structures and functions, and covalent drugs targeting these amino acids are effective in treating numerous diseases. There are 20 main types of natural amino acids, with different reactivities, in the proteins in the human body. In addition, the proteins and amino acids are affected by the spatial microenvironment, leading to significant differences in their spatial reactivities. The key in evaluating the reactivities of amino acids via ABPP is to select those with high reactivities. The core of the ABPP strategy is the use of chemical probes to label amino acid sites that exhibit higher activities in certain environments. The activity-based probe (ABP) at the core of ABPP consists of three components: reactive, reporter groups and a linker. The reactive group is the basis of the ABP and anchors the drug target via strong forces, such as covalent bonds. The reaction exhibits a high specificity and conversion rate and should display a good biocompatibility. Activity probes based on different amino acid residues have been developed, and the screening of amino acid activity combined with isotope labeling is a new focus of research. Currently, different types of ABPs have been developed to target amino acids and characterize amino acid reactivity, such as cysteine labeled with an electrophilic iodoacetamide probe and lysine labeled with activated esters. ABPP facilitates the discovery of potentially therapeutic protein targets, the screening of lead compounds, and the identification of drug targets, thus aiding the design of novel drugs. This review focuses on the development of ABPP methods and the progress in the screening of amino acid reactivity using ABPs, which should be promising methods for use in designing targeted drugs with covalent interactions.

蛋白质作为生命功能的执行者,其表达量、空间定位与结构差异是调控细胞功能活动的物质基础。随着人类蛋白质草图计划的初步完成^[1,2]^,研究者们发展了一系列用于蛋白质高通量、高灵敏度的定性与定量技术,并将其与蛋白质的结构稳定性等相结合,用于蛋白质翻译后修饰、结构蛋白质组学、蛋白质-蛋白质相互作用网络等多种生物学过程研究,进而为蛋白质参与生命活动过程的研究提供技术支撑和数据支持。其中,药物小分子与蛋白质之间的相互作用研究,将极大程度地推动新靶标的发现和原创药物的研制,进而提高临床治愈效率与国民的健康生活质量。然而,目前尚有超过85%的蛋白质被认为是不可成药的^[3]^,其主要原因是对蛋白质的功能信息缺乏了解,对蛋白质可以被药物分子靶向的空腔以及相应的反应活性位点缺乏研究,从而严重阻碍了新治疗策略的发展。

寻找新靶标是药物研发的重要环节,随着生物质谱技术的发展,基于化学蛋白质组学分析药物或小分子化学探针与靶标蛋白的相互作用取得了长足进步,该技术挖掘潜在药物的靶点或现有临床药物的未知靶点,为药物研发提供数据支持。化学蛋白质组学可分为化学修饰蛋白质组学方法和非化学修饰蛋白质组学方法两大类,其中非化学修饰蛋白质组学方法包括细胞热位移测定(cell thermal displacement assay, CETSA)、热蛋白组学分析法(thermal proteome profiling, TPP)和限制性蛋白酶解法(limited proteolysis, LiP)等方法^[4]^。以上方法基于蛋白质与其配体结合后,通过靶标蛋白及其复合物对热、氧化和酶解等应激状态的改变进行确认,但其无法应用于与药物分子结合构象没有发生显著变化的靶标蛋白。化学修饰蛋白质组学包括亲和色谱法(affinity chromatography, AC)和基于活性的蛋白质组分析(activity-based protein profiling, ABPP)等方法。AC是化学蛋白质组学策略中较为经典的方法之一,主要应用于研究蛋白质与生物活性小分子或蛋白质与蛋白质之间的相互作用,但研究分子衍生物活性的不确定性和材料配体结合力的差异性以及非特异性吸附都将会干扰研究结果。基于ABPP方法已被广泛应用于蛋白质的结构和功能状态的研究。通过活性小分子直接钓取靶标蛋白,该方法能够评估活性小分子和靶标蛋白的结合状态。针对目前缺乏药物靶标蛋白的挑战,研究者设计了多种类型的亲电活性小分子,实现了在蛋白质提取物、完整细胞乃至活体等层次蛋白质反应活性的标记。在此基础上,利用蛋白质组学的定量技术实现药物靶标蛋白的发现及其作用区域的鉴定^[5]^。通过以上研究,不仅有助于了解蛋白质的结构与功能,并且有助于发现新的药物靶点和先导化合物,从而解决传统意义上不可成药蛋白质的问题(如图1所示)。人体内蛋白质中主要存在20种具有不同反应活性的天然氨基酸,并且同一蛋白质、同一类型氨基酸受到空间微环境(包括氢键相互作用、局部pH值、氧化还原条件或诱导效应)的影响,导致其空间反应活性存在显著的差异。如何基于ABPP方法进行氨基酸反应活性的评估,筛选出高反应活性的氨基酸成为其中的关键。本文将聚焦基于不同类型活性探针的ABPP技术在氨基酸反应活性筛选领域的研究进展,并对其技术发展和应用前景进行展望。

**图1:**
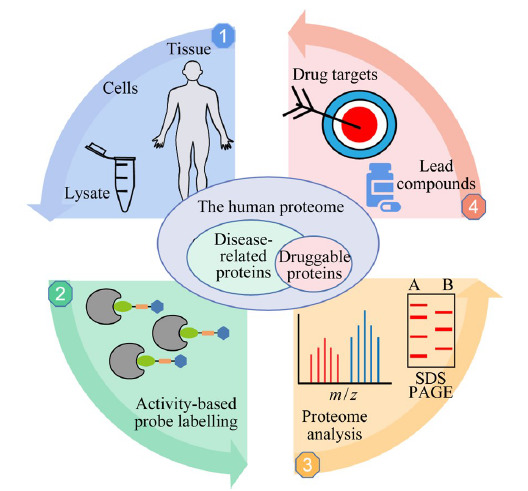
基于活性的蛋白质组分析解决不可成药蛋白质的问题

## 1 ABPP技术概况

ABPP是一种活性导向(主要包括反应活性、结合特异性等)的蛋白质组分析方法。1999年Cravatt教授等^[6]^首次提出这一概念,并将其用于蛋白酶的活性检测,包括丝氨酸水解酶^[7]^、蛋白激酶^[8,9]^、糖苷酶^[10,11]^、金属蛋白酶^[12]^以及氧化还原酶^[13]^等许多执行重要生物功能的蛋白酶家族^[14]^。并通过对酶活性的研究,筛选与疾病密切相关的关键酶,进而开发针对这些目标酶的选择性抑制剂用于疾病治疗^[15]^。

随着质谱技术的发展和定量蛋白质组学技术的深入,ABPP方法已成功用于药物靶标蛋白筛选等研究,成为蛋白质鉴定和表征功能的关键技术,其主要包括基于ABPP的直接富集和基于竞争性的ABPP间接富集两种策略(如图2所示): 1)基于ABPP的直接富集策略,首先通过反应基团将探针与靶标蛋白共价标记,后续利用点击化学将探针与报告基团(包括荧光、亲和富集等基团)相连接,再通过荧光光谱、质谱等技术进行标记蛋白的检测与鉴定,表征标记氨基酸的反应活性,进而挖掘更多的药物靶标蛋白。其中关键的是,探针以化学键的方式“冻结”小分子与靶标蛋白之间的相互作用,共价键连接可以实现低亲和力靶标蛋白的鉴定^[16]^。该策略具有富集简单、背景干扰低等优势,然而其面临着探针化合物化学修饰步骤繁琐、应用范围受限等挑战;2)基于竞争性的ABPP间接富集策略,首先利用化合物分子文库与蛋白质提取物共孵育,后续使用通用型工具探针分子进行蛋白质中非文库结合位点的标记,结合同位素定量分析,表征氨基酸与小分子化合物的配位性,挖掘潜在的药物先导化合物与其靶标蛋白。该策略无需对化合物进行化学修饰,而是基于通用型化学探针竞争标记策略间接进行不同小分子化合物靶标蛋白的筛选与鉴定,具有良好的通用性与分析通量。目前,该策略主要限于共价偶联药物及小分子药物的靶标蛋白研究^[17]^。综上所述,ABPP技术助力于潜在的治疗蛋白靶点的发现、先导化合物的筛选和药物靶点的识别,进而为原创药物的设计提供参考和帮助。其中,通用型化学探针的研制被誉为ABPP策略的“心脏”,其设计与研制历程将在下一节详细介绍。

**图2:**
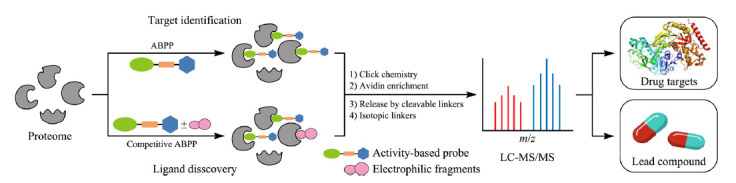
基于ABPP方法发现药物靶点和药物先导化合物

## 2 针对氨基酸反应活性的ABPP探针研制

如上所述,ABPP技术的核心是利用化学探针标记一类在某些特定环境中显示出更高活性的蛋白质位点^[18]^。因此,ABPP探针的选择是其成败的关键^[19]^。活性探针(activity-based probe, ABP)主要由反应基团、连接臂和报告基团三部分构成(如图3所示)。其中,反应基团是ABP探针的基础,其通过共价键等强亲和作用力实现药物靶标的锚定,其反应具有高特异性、高转化率的特征,并且反应需要具备良好的生物兼容性。报告基团也被称为报告标签,主要用于后续标记蛋白的可视化和富集检测。在标记蛋白的可视化研究中,荧光基团是一种常见的报告基团,主要有罗丹明、荧光素等荧光试剂^[20]^。此外,多数报告基团分子尺寸较大,存在着严重的空间位阻效应^[21]^。随着生物正交反应技术的发展,可视化报告基团逐渐被具有生物正交反应基团(包括炔基、叠氮基等)所取代。新型探针分子无需直接连接相对分子质量较大的报告基团,而是在蛋白标记后利用点击化学等反应引入生物素等报告基团,实现其可视化检测^[22⇓⇓-25]^。该策略既方便探针的合成,又避免了空间位阻影响探针的反应活性,同时为标记蛋白的富集和基于质谱等策略的检测引入可富集标签,为后续特异性的鉴定提供了保障。连接臂是反应基团与报告基团之间的桥梁,其通过调节化学反应活性与空间位阻,提高探针的稳定性与空间分布。通过以上3个部分的有机组合,进而实现ABP探针对靶标蛋白的高特异性富集与检测。

**图3:**
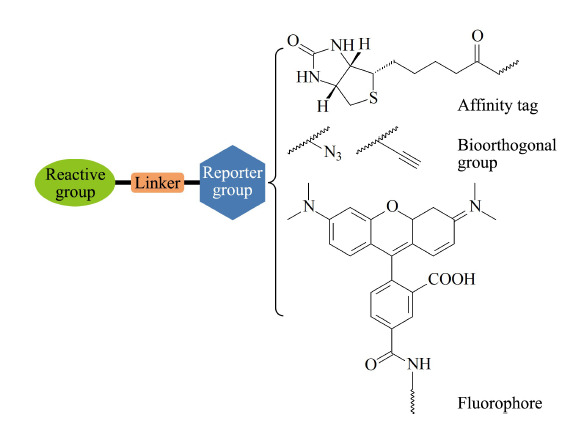
基于活性的化学探针

在基于ABPP策略的氨基酸反应活性表征研究方面,由于同一蛋白质的不同氨基酸所处的微环境不同,进而导致其反应活性存在显著差异。针对高反应活性氨基酸、蛋白质的表征,将为共价靶向药物的筛选提供新靶标,进而推动共价靶向类原创药物的研制,因此氨基酸反应活性的筛选已成为新的研究热点。目前,针对具有亲核/亲电活性的不同类型氨基酸,分别开发了对应的高效标记探针,进而用于氨基酸反应活性的表征。其中,以Cravatt研究团队为代表,针对半胱氨酸、赖氨酸等高亲核活性的氨基酸,发展了一系列具有不同亲电活性类型的探针,并将其应用于体外蛋白质提取物中氨基酸反应活性的表征^[26]^。近年来,基于ABPP策略的氨基酸反应活性研究扩展到其他具有低亲核活性的氨基酸,以下将以氨基酸类型进行分别详细介绍。

### 2.1 半胱氨酸的反应活性表征

半胱氨酸约占人类蛋白质中氨基酸含量的2.3%,虽然其丰度不高,但是其侧链的巯基基团具有高度的亲核性,并且其存在于超过97%的人源蛋白质序列中^[27]^。同时,半胱氨酸残基的位置具有高度保守性,其活性与蛋白质的生物化学功能紧密联系^[28]^。目前,半胱氨酸是许多临床共价靶向药物的反应靶点,如肺癌的二代和三代药物包括阿法替尼、奥希替尼等。

基于半胱氨酸的强亲核性,开发具有不同亲电性的ABP探针成为最佳的选择。亲电的碘代乙酰胺与半胱氨酸的巯基发生取代反应,使半胱氨酸发生烷基化。基于此反应原理,Cravatt等^[29]^研制了具有高反应活性的碘代乙酰胺-炔烃探针,并利用标记的炔烃进行点击化学反应,实现标记位点的富集与鉴定;研究发现半胱氨酸标记效率与浓度存在正相关性,进而发展了基于高、低浓度的半胱氨酸反应活性探测技术。进一步将其与稳定同位素标记技术相结合,发展了基于同位素标记定量的串联生物正交蛋白质标记表征技术(isoTOP-ABPP)。在此基础上,将其应用于乳腺癌等细胞系中半胱氨酸残基的反应活性表征,共定量到1082个半胱氨酸残基,其中包括350个具有高反应性半胱氨酸残基,这些位点与蛋白质的功能密切相关^[30]^。基于碘代乙酰胺-炔烃探针的探测技术,Backus等^[31]^构建了含有氯乙酰胺和丙烯酰胺的亲电小分子片段库,并在637种蛋白质中鉴定了758个配体半胱氨酸结合位点,分析结果发现所鉴定的蛋白质大部分尚未在Drugbank数据库中收录,其为后续新的药物靶标蛋白的发现和筛选提供了新的候选蛋白库。同时,发现GSTO1、RTN4、ACAT1、MLTK等与肿瘤高度相关的蛋白质对应的多个配体小分子,为靶向药物的研究提供了潜在的药物前体分子,进一步增加半胱氨酸的标记和鉴定覆盖深度,Bar-Peled等^[32]^将上述工作中发现的与半胱氨酸具有反应性的两个亲电小分子化合物用于非小细胞肺癌中潜在药物靶向活性半胱氨酸位点的高覆盖度检测,在6个非小细胞肺癌细胞系中实现了9700个半胱氨酸的高覆盖度定量,鉴定到约1100个可配位的半胱氨酸;同时发现NRF2的下游调控蛋白NR0B1中C274具有高反应活性,并被亲电的靶向片段药物分子进行共价修饰,从而破坏转录调控复合物的形成和抑制含有KEAP1突变的非小细胞肺癌的增殖,为肺癌靶向治疗研究提供了候选方案。在进一步的工作中,Vinogradova等^[33]^通过对富集连接臂与断裂基团进行改造,绘制了静止和刺激状态下原代人类T细胞中半胱氨酸反应性和亲电小分子相互作用图谱,鉴定到2283种蛋白质中3466个配体结合半胱氨酸,并且发现亲电化合物通过对蛋白酶的活性直接抑制以及降解等途径抑制T细胞活化,进而为获得性免疫的调控研究提供新维度。针对以上技术需要多步标记、操作繁琐的问题,Kuljanin等^[34]^发展了基于脱硫生物素碘乙酰胺探针和定量的串联质谱标签(tandem mass tags, TMT)技术相结合的一步“标记-富集”技术,实现高通量筛选半胱氨酸反应性片段库,共表征了超过8000个半胱氨酸残基反应性和285个亲电小分子片段的配位能力,实现了人源细胞蛋白中半胱氨酸活性位点的高覆盖度鉴定与活性筛选。

目前,针对半胱氨酸反应活性的研究主要依赖于碘代乙酰胺-炔烃探针,其具有反应速率快、标记活性高的优势。同时,针对碘代乙酰胺-炔烃探针的细胞毒性高、难以实现活细胞层次标记的难题,Abo等^[35,36]^开发了光笼基团保护的溴、碘取代亲电探针,其与碘代乙酰胺-炔烃探针的反应原理相同,溴离子、碘离子与半胱氨酸的巯基发生亲电取代反应。但该探针和碘代乙酰胺类非保护探针相比毒性显著降低,实现了活细胞中半胱氨酸的标记。另一方面,烯丙基酮类中的烯键易与巯基等发生迈克尔加成反应,该反应条件温和、结构稳定,不易发生光分解等副反应,因此成为共价抑制剂设计中普遍采用的反应弹头。如上所述,碘代乙酰胺-炔烃探针广泛用于半胱氨酸反应活性探测,烯丙基酮类分子则被用于共价抑制剂的开发,这二者的反应原理不同,导致半胱氨酸与二者的反应活性存在差异,进而容易引起药物设计的偏差。因此,发展基于烯丙基酮类探针的半胱氨酸反应活性表征技术成为新的研究方向。Yu等^[37]^开发了4-乙酰氧基环戊烯酮探针,其通过迈克加成反应标记蛋白中的半胱氨酸,并进一步通过弱碱性条件下的*β*-消除反应实现了半胱氨酸的可逆标记。*α*,*β*-不饱和烯醛与半胱氨酸的巯基具有更高的反应活性,Zhang等^[38]^用4-羟基壬烯醛(HNE)探针标记半胱氨酸,进而利用醛与羟胺之间的反应,结合多重稳定同位素标记-亲和纯化(SiLAP)策略在肝细胞系中鉴定到626个半胱氨酸修饰位点。以上策略初步展示了烯丙基酮/醛类探针在半胱氨酸反应活性表征方面的应用潜力。然而,以上两种探针分子存在较大的空间尺寸位阻效应,导致半胱氨酸的检测覆盖度较低。因此,低空间位阻、高反应活性的烯丙基酮/醛类探针仍是半胱氨酸活性探测研究的新方向。

### 2.2 赖氨酸的反应活性表征

在蛋白质氨基酸中,赖氨酸不仅具有较高的丰度(占人类蛋白质中的5.9%),而且它的侧链伯胺具有一定的亲核性,容易与探针发生共价生物偶联^[39]^。更重要的是,赖氨酸通常是许多蛋白质的功能位点,比如是酶活性中心位点或者位于蛋白质配体的结合口袋中,进而介导蛋白质及其配体之间的相互作用。同时,赖氨酸是生物体内最重要的翻译后修饰的位点,包括酰化修饰、甲基化修饰、泛素化修饰等翻译后修饰类型,其直接调控了细胞的表观遗传、细胞的发育和增殖等重要生物学过程^[40]^。因此,基于其重要的生理功能,赖氨酸被认为是最有潜力的共价靶向药物结合位点。针对赖氨酸靶向药物的研究也日益引起关注,如PI3K激酶活性位点中的赖氨酸是天然产物渥曼青霉素的靶标,开发出了PI3K的共价抑制剂,并已经进入非小细胞癌、去势抵抗性前列腺癌与胶质母细胞瘤的临床试验^[41,42]^。

羧基活化产生的活性酯是目前标记赖氨酸的主要反应基团,该基团可与赖氨酸的氨基发生取代反应,现已开发了一系列活性酯化学探针用于赖氨酸的选择性标记。Ward等^[43]^开发了基于商品化的羟基琥珀酰亚胺酯-炔烃(NHS)探针标记小鼠肝脏组织中赖氨酸残基的方法,该方法鉴定到1639个赖氨酸残基,且31%是在UniProt网站注释的功能位点,包括结合活性中心、钙离子结合位点和翻译后修饰位点等。基于NHS的反应条件温和,具有良好的生物兼容性,合成基于NHS-酯的化合物用于靶蛋白中的赖氨酸的筛选,如二氢嘧啶脱氢酶(Dpyd)中的K497和乙醛脱氢酶2(Aldh2)中的K211等,以上位点为靶向药物的设计提供了候选靶标。然而,NHS标记试剂存在易水解、交叉性副反应等问题。针对这一挑战,Hacker等^[44]^研制了磺基四氟苯酯(STP)探针用于赖氨酸反应性与配位性的表征,利用该策略鉴定了人源蛋白质组中超过9000个赖氨酸残基,并筛选出310个高反应性赖氨酸残基。在此基础上,研究者合成了51个活性酯的小分子化合物,将其用于赖氨酸残基与小分子配体的相互作用探测,结果表明小分子与赖氨酸之间的共价标记有效抑制了靶标蛋白质的功能;将其用于SIN3A-TGIF1通路的阻断,实现了针对三阴性乳腺癌靶向干预研究。Abbasov等^[45]^进一步扩展了赖氨酸反应的小分子化合物库,表征了180个亲氨基化合物与14000个赖氨酸的反应性,为具有靶向赖氨酸位点的共价药物的开发提供了更多的信息。

上述探针主要基于赖氨酸侧链氨基与活性酯的取代反应,该反应具有条件温和、反应速率高等优势。然而,活性酯存在尺寸大、易水解等问题,不利于共价靶向抑制剂的研制。针对这一挑战,Tang等^[46]^开发了可调胺反应性亲电试剂(TAREs)作为赖氨酸的共价探针,该探针具有稳定性好、毒性低等优势,在共价抑制剂的开发方面具有应用潜力。醛基与伯胺发生席夫碱反应,其产物易发生降解。对醛基的取代基进行调控,结合蛋白质的空间限域效应,有助于形成稳定的加合物。Quach等^[47]^基于羰基硼酸与赖氨酸氨基的反应,设计了第一个靶向赖氨酸的BCR-ABL (breakpoint cluster region Abelson leukemia virus)可逆共价激酶抑制剂,该抑制剂在体外实验中展示了优异的抑制效果,但是在活细胞层次抑制效果较差。在此基础上,他们进一步开发了基于水杨醛的可逆共价探针用于ABL激酶催化域赖氨酸的靶向修饰^[48]^,该探针在治疗慢性粒细胞白血病(CML)研究中,展现出良好的细胞活性抑制效果。邻硝基苄醇衍生物在光激活条件下会产生邻亚硝基苯甲醛,其与伯胺发生环化反应生成吲唑酮类化合物^[49,50]^,该反应具有标记效率高、反应可控等优势。Chen等^[51]^将此反应与化学交联技术相结合,开发了具有赖氨酸选择性的遗传编码蛋白质光交联剂。利用紫外光照激活,该光交联剂可以在体外和活细胞中检测蛋白-蛋白相互作用。在进一步的工作中^[52]^,将该光交联剂引入生物正交反应基团,开发了基于光诱导的点击化学反应,在活细胞层次实现对赖氨酸的特异性标记。该策略具有反应可控的优势,在活细胞内的标记具有重要应用潜力。综上所述,随着醛基反应、光标记等可控反应的引入,赖氨酸的活性标记反应逐渐由细胞裂解液转到活细胞层次,这极大地推动了赖氨酸作为共价键的靶向药物的研制。

### 2.3 酪氨酸的反应活性表征

酪氨酸在人类蛋白质中占3%,多处于蛋白质表面,是修饰蛋白质的重要位点^[53,54]^。酪氨酸具有很重要的生物学功能,比如参与蛋白质磷酸化,酶底物之间的结合以及蛋白-蛋白相互作用等^[55]^。目前,已报道了多种靶向酪氨酸的共价抑制剂^[56]^。如Hett等^[57]^利用磺酰氟共价抑制剂特异性靶向mRNA脱壳酶DcpS中的酪氨酸残基,用于治疗脊髓性肌萎缩症。因此,酪氨酸反应活性的探测也至关重要。

在酪氨酸的标记方面,Joshi等^[58]^利用醛和带电子的苯胺生成亚胺中间产物标记酪氨酸,该反应条件较温和。但是,在后续的研究中发现该反应特异性不强,存在色氨酸修饰等副反应的干扰^[59]^。Chen等^[60]^用芳基氟硫酸盐探针标记酪氨酸,可以靶向脂质结合蛋白iLBPs配体结合位点中的保守酪氨酸残基,但是该反应的特异性也较差。Hahm等^[61]^通过硫-苯三唑交换反应(SuTEx)实现对酪氨酸位点的特异性标记,用三唑基团替换氟原子增加对酪氨酸的特异性。鉴定到了3000个蛋白质中的8000多个酪氨酸位点,这些位点广泛分布在酶以及蛋白质相互作用的结构域中。通过稳定同位素细胞培养标记(SILAC)定量了2400个酪氨酸残基,筛选出127个高反应性酪氨酸残基,这些位点与酪氨酸磷酸化的程度呈负相关。Brulet等^[62]^通过进一步改变SuTEx中的离去基团和加成基团来调节对酪氨酸的反应活性和位点选择性,筛选出300多个可配位的酪氨酸位点,并发现基于SuTEx探针设计的JWB142化合物可以选择性地靶向金属肽酶DPP3中的Y417位点来抑制靶标活性,JWB198化合物靶向GSTP1的Y8位点来抑制其活性,这一技术为靶向酪氨酸的先导化合物研究提供候选分子。酪氨酸的侧链苯酚可以与重氮盐发生偶联偶氮反应^[63]^, Sun等^[64]^通过改变重氮盐苯环上的取代基,增强其对酪氨酸的反应性。该反应的标记产物可以进一步用亚代硫酸钠释放,所以该探针标记引入的标签尺寸非常小,实现了超过5000个酪氨酸位点的鉴定。结合TMT标记筛选到75个高反应性酪氨酸残基,这些高反应性位点大多数位于活性功能蛋白质中,包括催化活性、激酶结合活性和水解酶活性等蛋白质。通过以上策略,初步实现对酪氨酸的选择性标记,并利用反应的可逆性,显著降低质量标签的相对分子质量,有助于后续的质谱鉴定,进而提高标记酪氨酸的鉴定覆盖度,为其活性筛选提供了技术支持。

### 2.4 其他低亲核性氨基酸的反应活性表征

除了上述强亲核性的氨基酸残基外,还有许多低亲核性的氨基酸,主要包括谷氨酸、天冬氨酸、甲硫氨酸和组氨酸等。目前,针对其活性的研究也取得了重要进展。

谷氨酸和天冬氨酸是酸性氨基酸,含有羧基基团,既是氢键的供体,又是受体^[65,66]^。这一特性大大增加了其特异性标记的难度。Li等^[67]^研制了二芳基四唑光交联剂,在紫外线照射下,可以实现特异性标记谷氨酸和天冬氨酸。在此基础上,进一步设计了四唑骨架的小分子探针光交联探针库^[68]^,实现了癌症标志物膜联蛋白A2(ANXA2)的高特异性标记。Bach等^[69]^通过可见光活化的2,5-二取代四唑探针结合ABPP技术表征细菌蛋白质组中的天冬氨酸和谷氨酸的反应活性,在裂解液和活细胞层次共定量了近9000个天冬氨酸和谷氨酸残基。以伍德沃德氏试剂K(WRK)和异噁唑盐作为配体化合物,表征了44个天冬氨酸和谷氨酸与配体化合物的相互作用。这一技术填补了羧基氨基酸活性表征的空白,促进了针对羧基共价抑制剂的开发。Ma等^[70]^开发3-苯基-2*H*-吖丙因化学探针,在裂解液和活细胞层次标记天冬氨酸和谷氨酸,鉴定到1128个谷氨酸和549个天冬氨酸位点,且生成的加合物*N*-苯基乙酰胺具有高稳定性。该团队结合isoTOP-ABPP技术筛选到与恶性肿瘤密切相关的靶标蛋白,如胰腺癌的生物标志物BASP1蛋白,食管鳞状细胞癌的生物标志物PTMA蛋白等,为相关肿瘤的早期高灵敏度检测和后续的靶向治疗提供候选蛋白质分子。综上,结合光活化的四唑探针和3-苯基-2*H*-吖丙因探针的开发,现已在裂解液和活细胞层次实现天冬氨酸和谷氨酸的活性表征。然而,目前针对天冬氨酸和谷氨酸的探针开发仍是有限的,尤其是具有标记特异性的探针仍有待进一步拓展。

甲硫氨酸与半胱氨酸都是含硫氨基酸,但是甲硫氨酸的亲核性非常弱,并且具有一定疏水性^[71,72]^。与半胱氨酸类似,甲硫氨酸在蛋白质中的丰度比较低,仅有2%左右。甲硫氨酸作为蛋白质翻译的起始氨基酸,属于生物体的必需氨基酸,具有高度保守性,并且对体内的氧化-还原环境高度敏感。然而,甲硫氨酸残基多处于蛋白质内部,难以进行精准的靶向^[73]^,针对甲硫氨酸化学探针的开发仍是一个巨大挑战^[74]^。Kharenko等^[75]^通过靶向甲硫氨酸开发与多种癌症相关的溴结构域蛋白-4(BRD4)的共价抑制剂,显示了开发以甲硫氨酸为靶点的共价抑制剂的应用前景。Chang等^[76]^利用氧化还原化学标记(ReACT)策略标记蛋白质中的甲硫氨酸,通过氧氮丙啶试剂与硫醚形成亚磺酰亚胺标记,在生理条件下标记了235个甲硫氨酸残基,进一步将该策略与isoTOP-ABPP技术相结合,筛选到111个具有高反应性的甲硫氨酸残基,这些位点包括糖酵解代谢途径中心烯醇化酶中的3个高反应性甲硫氨酸残基。尤其值得注意的是,靠近活性位点高度保守的171位点对烯醇化酶功能的氧化还原调节至关重要,发现171位点突变的菌株对氧化应激诱导的细胞死亡具有更强的抵抗力。综上,基于氧氮丙啶开发的ReACT策略可以实现对甲硫氨酸的活性表征,但是现有针对甲硫氨酸的化学探针种类仍比较单一,尚难以实现甲硫氨酸功能位点图谱的高覆盖度检测,有待新的化学标记反应及探针的引入。

组氨酸在人类蛋白质中约占2%,是p*K*_a_值唯一接近7的氨基酸^[77]^。组氨酸的侧链为咪唑基团,在生理环境中既能接受质子又能提供质子,发挥着质子传递的作用。同时,组氨酸占据超过1/5人源酶活性中心,在蛋白质中发挥多重作用^[78]^,包括与金属离子配位、质子受体和酸碱催化等^[79,80]^。目前,组氨酸磷酸化修饰已被报道与恶性肿瘤疾病密切相关^[81⇓-83]^。Zhang等^[84]^发现白喉毒素催化真核翻译延伸因子2 (eEF2)发生ADP核糖基化修饰,组氨酸是白喉疾病的修饰靶点,通过靶向组氨酸的小分子可以有效阻断白喉疾病,是白喉疾病理想的治疗靶点^[85]^。然而,组氨酸侧链的反应活性极低,常规亲核试剂难以与其形成稳定的共价键,对于组氨酸化学探针的开发仍处于起步阶段。Li等^[86]^利用环氧化物实现组氨酸残基标记,并研制了基于环氧化物的荧光探针,用于测定人血清中的组氨酸探测。然而,该反应条件相对苛刻,在生理条件下难以实现对组氨酸的标记及活性探测。烯烃可以和组氨酸的侧链咪唑基团发生迈克尔加成反应,Joshi等^[87]^基于此反应原理研发环己烯酮特异性标记组氨酸,但该反应的反应速度较慢,难以实现组氨酸的快速标记。Jia等^[88]^受组氨酸磷酸化翻译后修饰的启发,合成了一系列关于膦亲电试剂,在碱性条件下实现了组氨酸的快速标记。该化学探针可以实现组氨酸的标记,但是尚难以实现生理条件下组氨酸的快速标记,进而难以在复杂样品中对组氨酸活性进行全局分析。我们在近期的工作中发展了基于*α*,*β*-不饱和烯醛的组氨酸标记反应,初步实现了生理条件下组氨酸的高效标记,目前工作还处在进一步完善中。

综上所述,虽然已有部分化学探针可以靶向天冬氨酸、谷氨酸、甲硫氨酸和组氨酸等亲核性较弱氨基酸^[89,90]^,但是针对亲核较弱氨基酸的探针开发仍是很大的挑战,尤其是尚缺乏对组氨酸活性进行全局分析的探针。因此,亟需开发与ABPP方法兼容的新型反应弹头,探索新的药物靶点和药物先导化合物。

## 3 总结与展望

基于化学探针和活性蛋白质组学相结合技术评估氨基酸侧链的反应活性,进而筛选新的药物靶点及其先导化合物,可以助力于共价抑制剂药物的开发^[91]^。目前研究的化学探针主要靶向半胱氨酸、赖氨酸等具有强亲核性的氨基酸,关于亲核性较弱的氨基酸(如组氨酸、甲硫氨酸和天冬氨酸)等仍需要开发新的化学探针,进而实现对更多不可成药蛋白质的共价键靶向调控研究。未来ABPP的发展可能还会涉及其他的小分子,比如RNA、DNA中的活性位点,帮助开发疾病治疗新策略。同时,ABPP蛋白组学富集方法需要在化学探针上修饰炔基或叠氮基团,进而基于点击化学引入生物素等反应探针进行靶标蛋白的鉴定。然而,针对基于氨基酸反应活性的探测反应,最关键的是修饰肽段的鉴定。目前,基于点击化学的策略需要多步富集,容易引入大质量标签,不利于标记肽段的鉴定。如何发展针对修饰肽段的一步捕获与可逆释放技术成为其中的关键。我们课题组前期发展了基于可逆的酰肼化学富集的*O*-GalNAc和*O*-GlcNAc糖肽富集新方法^[92⇓-94]^,其主要是通过醛基与酰肼、羟胺之间的可逆反应实现了标记肽段的可逆富集。与常规的点击化学策略相比,该策略具有反应效率高、引入质量标签小等优势,利于标记肽段的鉴定。因此,可逆的酰肼化学作为一种新的富集方法,一步反应即可实现标记与富集,在荧光和质谱的检测方面都具有突出优势。同时,其他基于可逆反应的新型氨基酸标记策略的引入,也必将推动氨基酸反应活性表征技术的发展,进而为靶向药物的研究加入新的活力。
